# Bredigite-CNTs Reinforced Mg-Zn Bio-Composites to Enhance the Mechanical and Biological Properties for Biomedical Applications

**DOI:** 10.3390/ma16041681

**Published:** 2023-02-17

**Authors:** Hongwei Zhang, Abbas Saberi, Zahra Heydari, Madalina Simona Baltatu

**Affiliations:** 1 School of Mechanical Engineering, Xijing University, Xi’an 710123, China; 2 Department of Materials Engineering, South Tehran Branch, Islamic Azad University, Tehran 1777613651, Iran; 3 School of Electrical and Computer Engineering, College of Engineering, University of Tehran, Tehran 1439957131, Iran; 4 Department of Technologies and Equipments for Materials Processing, Faculty of Materials Science and Engineering, Gheorghe Asachi Technical University of Iaşi, Blvd. Mangeron, No. 51, 700050 Iasi, Romania

**Keywords:** magnesium, Br-CNTs nanofillers, mechanical properties, degradation, antibacterial activity, biocompatibility

## Abstract

Magnesium (Mg) and its compounds have been investigated as biodegradable metals for bone implants. However, high corrosion rates and low bioactivity that cause loss of mechanical properties are factors that have limited their biomedical applications. The purpose of this work is to remedy the weaknesses of the Mg–Zn (MZ) alloy matrix. For this purpose, we have synthesized Mg-based composites with different concentrations of bredigite (Br; Ca_7_MgSi_4_O_16_)–carbon nanotubes (CNTs) using mechanical alloying and semi-powder metallurgy processes with spark plasma sintering. Then, we studied the effect of the simultaneous addition of Br-CNTs on in vitro degradation, as well as its effect on the composites’ mechanical and antibacterial properties. Increases of 57% and 72% respectively were observed in the microhardness and compressive strength of the MZ/Br-CNTs composite in comparison to the MZ alloy. In addition, the rate of degradation of Mg-based composites in simulated body fluids (SBF) was almost 2 times lower. An assessment of antibacterial behavior disclosed that the simultaneous adding of Br-CNTs to Mg can meaningfully prevent the growth and invasion of *E. coli* and *S. aureus*. These research findings demonstrate the potential application of MZ/Br-CNTs composites to implants and the treatment of bone infections.

## 1. Introduction

The growing request for the use of implants has led to the need for implant modification and improvement [1]. At the same time, magnesium (Mg) and its alloys have been considered for use in the field of biomaterials due to their biocompatibility and biodegradable properties [1,2,3]. Mg has a density close to that of bone and is absorbed by the body [3]. Mg is of interest for orthopedic applications due to its relatively low cost, high specific strength, and near-bone elastic modulus, which provides stress shielding and allows uniform distribution of tissue stress [4,5,6]. Moreover, the biodegradability of Mg obviated the need for secondary surgery after bone healing [5]. Mg is the fourth most abundant ion in the human body; a 150-pound person stores about 1 mole of Mg, half of which is in bone tissue. Therefore, Mg is an essential and necessary element of the body’s metabolism [7,8,9,10]. Naturally, the rate of Mg degradation and reduction in mechanical features is faster than the rate of bone tissue regeneration in the body’s biological environment [6,7,10,11].

Nevertheless, alloying is a powerful approach to enhance the characteristics of Mg [4,12,13]. When picking an alloying element as the base alloy, it must be ensured that the element is not toxic in the body [12,14]. Therefore, according to various studies, the choice of alloy elements available is limited to a few nontoxic essential elements [9,15,16]. Zinc (Zn) is also one of the most abundant elements required for the body’s biological processes, and the body must routinely provide the minimum amount of this element [17]. It is kept in the muscles and also exists in blood cells, retinas, bones, skin, kidneys and the liver. Zn is essential for cell growth and the body’s bones. Researches have revealed that Zn promotes osteoblast adhesion of bone cells, as well as cell proliferation and differentiation [17]. Koç et al. [18] revealed that increasing the Zn portion of a Mg–Zn alloy decreased the grain size and improved the mechanical features, hardness and corrosion resistance of the alloy in laboratory environment [18].

Among the most common approaches to attain the supreme characteristics of Mg is to make composites with suitable nanofillers [19]. Carbon nanotubes (CNTs) are attracting a great deal of attention as nanofillers due to their unique properties, such as high Young’s modulus (1TPa) and strength (30 GPa) [20]. CNTs are one of the best reinforcements for Mg matrix composites [20,21]. Ding et al. [22] for example, added CNTs to Mg in powder metallurgy and observed significant improvements in mechanical properties compared to pure Mg [22].

Although these reinforcing materials may cause biosafety issues and enhance the antibacterial behavior of Mg-based alloys when used for biological applications, to date the research on CNTs as reinforcement in Mg alloys has investigated their mechanical characteristics. Among calcium silicate bioceramics, bredigite (Br; Ca_7_MgSi_4_O_16_) is one of the most well-known. It is a calcium silicate magnesium compound with an orthorhombic structure [11], and is likely to release silicon (Si) ions, thereby inducing precursor cell growth and cell differentiation. Si also causes the development and growth of bone and connective tissue, the deficit of which leads to abnormal bone formation [11,23]. Bredigite bioceramics also possess exceptionally high apatite properties, enabling tissue growth and bio-stabilization, resulting in stability and rapid stabilization of surrounding bone implants, accelerating healing rates [11,23].

Review of studies reveals that no attempts have been made to study the attendant effects of Br-CNTs on Mg-based composites. Here, we studied the potential of using Br-CNTs nanosystems during the manufacturing process for the first time to improve the mechanical and antibacterial behaviors of Mg-based composites. Mechanical alloying (MA) and semi-powder metallurgy (SPM) techniques, along with spark plasma sintering (SPS), were used to create MZ/Br-CNTs composites containing varying amounts of Br-CNTs fillers, and their mechanical, corrosion and biological behavior were investigated.

## 2. Materials and Methods

### 2.1. Raw Materials

Mg (99.5%, <10 µm) and Zn (99.9%, <3 µm) powders supplied by Merck Co. were alloyed by a mechanical alloying process in the argon atmosphere of a planetary ball mill device. The powder encapsulated in a sealed 120 mL steel container was ground at 300 rpm for various times. A mixture of bullets (Ø = 10 mm ball with 4.07 g mass and Ø = 20 mm ball with 32.65 g mass) was filled in the container (the ratio of bullets to powder was about 20:1).

CNTs (30–50 nm in diameter, 10–25 µm in length, 95% purity) and calcium silicate bioceramic powder, bredigite (Ca_7_MgSi_4_O_16_) with particle size ≤ 100 nm were used as reinforcements. After mechanical alloying through SPM, Mg composite reinforced by CNTs and Br nanoparticles was developed in this study.

**Figure 1:**
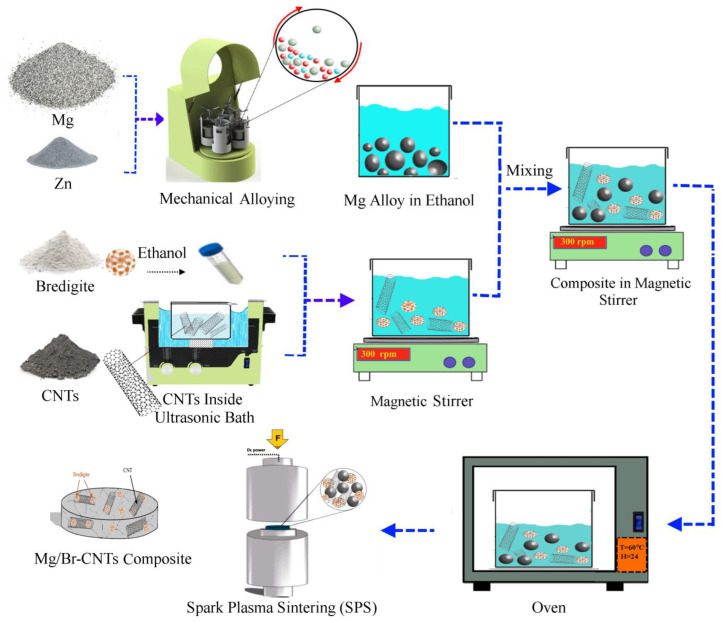
Schematic representation of the fabrication process of MZ/Br-CNT composites using SPM, and SPS methods.

To make MZ/Br-CNTs nanobiocomposite powder, Mg-Zn alloy powder obtained from mechanical alloying was poured in pure ethanol solution. Prior to this transition, CNTs were put in an altoarsenic bath to prevent CNTs adhesion. Then, Br nanoparticles and CNTs were mixed with a weight ratio of 10:1, and 0-0, 5-0.5, 10-1, and 15-1.5 (wt.%) of Br-CNTs were mixed with Mg alloy (Table 1) in a magnetic stirrer for 2 h at 300 rpm. The composite powder was then kept in the oven under controlled conditions for 24 h. Composite powders were labeled as MZ, BC1, BC2 and BC3, respectively. Finally, the composite powders were baked in an SPS chamber at a temperature of 570 °C and a pressure of 40 MPa for 10 min. The process scheme is shown in Figure 1.

### 2.2. Microstructres and Mechanical Characteristics

The microstructure and composition distribution were investigated using a scanning electron microscope (SEM, QUANTAFEG250, FEI, Lincoln, NE, USA) and energy dispersive spectroscopy (EDS, JSM-5910LV, JOEL Ltd., Akishima, Japan) as well as transmission electron microscopy (TEM; H-800, Hitachi, Tokyo, Japan). The phase and crystal structure features of samples were studied via X-ray powder diffraction (XRD) pattern (D8 Advance X-ray Diffractometer (equipped with a Cu Kα source of 1.5405 Å wavelength, scan rate 8°/min, XRD, D8 Advance, Brucker, Karlsruhe, Germany)). Moreover, the functional groups of the CNTs were determined using a Raman spectrometer (Takram P50C0R10 with a laser wavelength of 532 nm). A contact angle test was carried out on the surface of the sample according to the sessile drop technique (Dataphysics OCA 15) in air and at room temperature with a droplet size of 10 mL to determine the final wettability. A contact angle value for each composite was calculated by averaging three measurements. Compressive strength of cylindrical composite materials (diameter = 10 mm, height = 15 mm) for all samples was evaluated by pressing them at room temperature at a speed of 2 mm/min and a load of 10 kN according to the ASTM-E9 standard. Vickers microhardness test method (LECO M-400) was applied with a force of 300 g to find the microhardness of the composites. To obtain the results, we analyzed 5 different points from each sample. 

### 2.3. Degradation Rate Evaluation

The samples were treated in a pH 7.4 solution at a temperature of 37 °C according to the ASTM-G31-72 standard [24]. To study and measure degradation kinetics, composites containing varying amounts of reinforcement were placed in SBF solution for 2 weeks. Weight loss and hydrogen release rates were monitored every 12 h while immersion in SBF (*n* = 3) was ongoing.

To investigate the degradation of the implant, the rate of hydrogen release in the degradation process was tested. Briefly, released hydrogen bubbles were collected in a funnel and the SBF volume change was measured using a standard calibrated burette attached to the funnel. The corrosion products of the degradation process were tested using EDS and SEM. The weight loss (*W_loss_*) of the composites was also calculated using the following equation:

(1) $$W_{loss}=\frac{W_{1}-W_{2}}{W_{2}}\times 100$$

Here, *W*_1_ and *W*_2_ indicate the weight of the sample before and after immersion, respectively. The rate of degradation was quantitatively checked in Equation (2):

(2) $$\left({\mathrm{mm}\;h}^{-1}\right)=\frac{W_{loss}}{A\times T\times \rho }$$

In this formula, *A*, *T* and *ρ* are, respectively, surface area (mm), immersion time (h), and theoretical density (g/mm^3^).

### 2.4. Antibacterial Activity Evaluation

To investigate the biological activity of each sample (*n* = 3), the antibacterial behavior against Gram-negative (*E. coli*) and Gram-positive (*S. aureus*) bacteria was measured by disc diffusion method. To perform this test, a sterile swab was inserted into the sample, the bacterial suspension was washed (press the swab against the side of the tube), and the medium was viewed as a culture. Samples were placed in a 37 °C incubator for 1 day.

Gentamicin discs were used as the agar antibiotic. If the sample has antibacterial activity, this can be discerned through an inhibition area (IA) around the sample.

### 2.5. Biocompatibility Assessment

Prepared composites were sterilized under UV irradiation for at least 2 h before the cell test. A 3-(4,5-Dimethylthiazol-2-yl)-2,5-diphenyltetrazolium Bromide (MTT) test was employed to check the cell proliferation rate. First, 104 cells were poured into a 96-well cell culture plate and placed in a 37 °C incubator for 24 h to allow the cells to adhere to the bottom of the plate. Extracts taken from each sample were added to culture wells and cells were kept near these extracts for an additional 24 h. After that, the medium was removed and 100 μL of MTT at a concentration of 0.5 mg/mL was added to each well. After 4 h, the solution on the cells was removed and isopropanol was poured into each well to solve the metamazan crystals formed inside the cells. After 30 min the intensity of the color produced at a wavelength of 545 nm was calculated. Wells with more cells showed higher optical density than wells with fewer cells. The following relationships can be used to identify wells with the highest number of cells and compare them to control samples. Viable cell percentage = (average optical density sample)/(control average optical density sample). Cells cultured with control culture medium were considered the control. For each sample, there were three repetitions and in each repetition 5–10 houses from 96 plates were considered. To study the effect of tetracycline on early osteogenic differentiation of M-G63 cells, ALP activity assays were performed on days 3 and 7. 104 cells per ml were seeded and plated separately in 24-well plates. Cells were cultured for several days at 37 °C in a controlled atmosphere of 5% CO_2_.

### 2.6. Statistical Analysis

The analysis results were presented as mean ± standard error (SE) and momentous differences were tested using Sigmaplot software, version 12.0 (Systat software lnc., San Jose,CA, USA), with *p*-values of 0.05 (*), 0.01 (**), and 0.001 (***) data.

## 3. Results and Discussions

### 3.1. Microstructures

**Figure 2:**
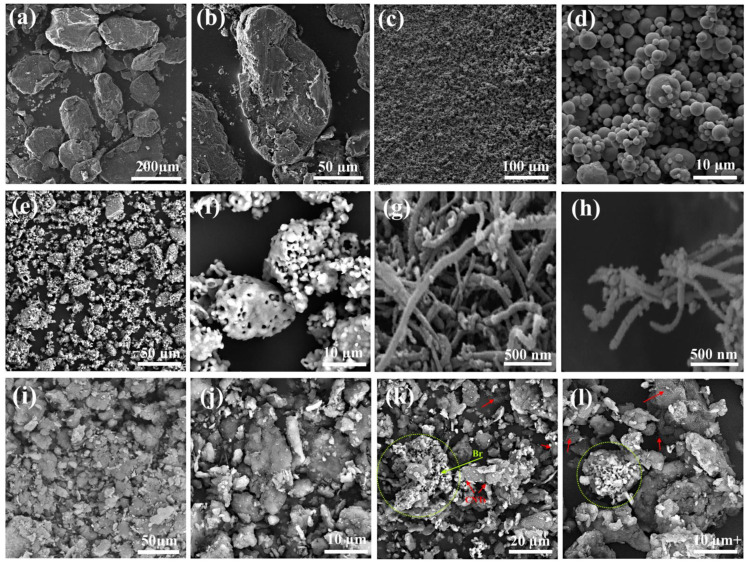
SEM images, at different magnification of (**a**) Mg ×300, (**b**) Mg ×1000 (**c**) Zn ×500, (**d**) Zn ×5000, (**e**) bredigite ×1000, (**f**) bredigite ×5000, (**g**) CNTs ×60,000, (**h**) CNTs ×60,000, (**i**) MZ ×1000, (**j**) MZ ×5000, (**k**) MZ/Br-CNTs ×2000 and (**l**) MZ/Br-CNTs ×3500 powder composites.

The SEM micrographs in Figure 2 show the morphology of pure powders related to (a,b) Mg, (c,d) Zn, (e,f) bredigite, and (g,h) CNTs that have not yet been subjected to mechanical or chemical stress. For milling and mechanical alloying, 97 wt% and 3 wt% of pure Mg and Zn powder were added to the grinding chamber. Powder particles are constantly subjected to expansion, cold welding, fracture and re-welding during high-energy milling. The result of this process is the formation of alloy powders (MZ) with new surfaces that are the result of the fusion and penetration of particles into each other and the creation of larger particles (Figure 2i,j). Figure 2k,l also show Mg-based composite powders (MZ/Br-CNTs) that were produced by adding bio-ceramic reinforcements of bredigite and CNTs by SPM method, as indicated by arrows.

**Figure 3:**
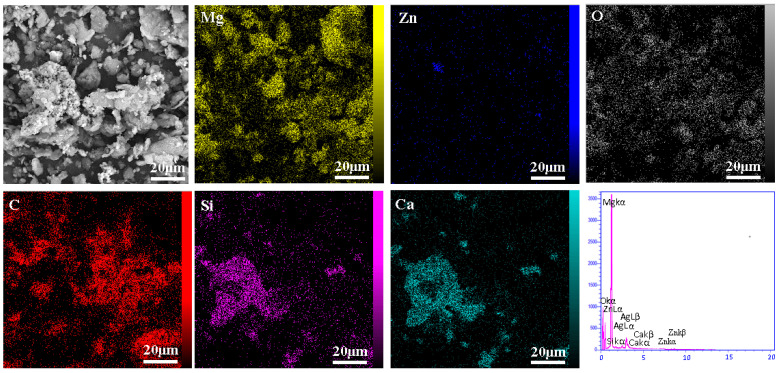
SEM and EDS (map and point) spectra of composite powders.

According to Figure 3, SEM images and EDS map spectra confirm that the composite powder contains Mg, Zn, O, C, Si and Ca. A dense overlapping mass of Ca and Si is clearly visible, indicating the presence of a bioceramic reinforced bredigite (Ca_7_MgSi_4_O_16_) bound to a Ca-containing magnesium silicate compound. Although the amount of CNTs is small, they are evenly distributed in the Mg field and can be seen as a reinforcing material.

**Figure 4:**
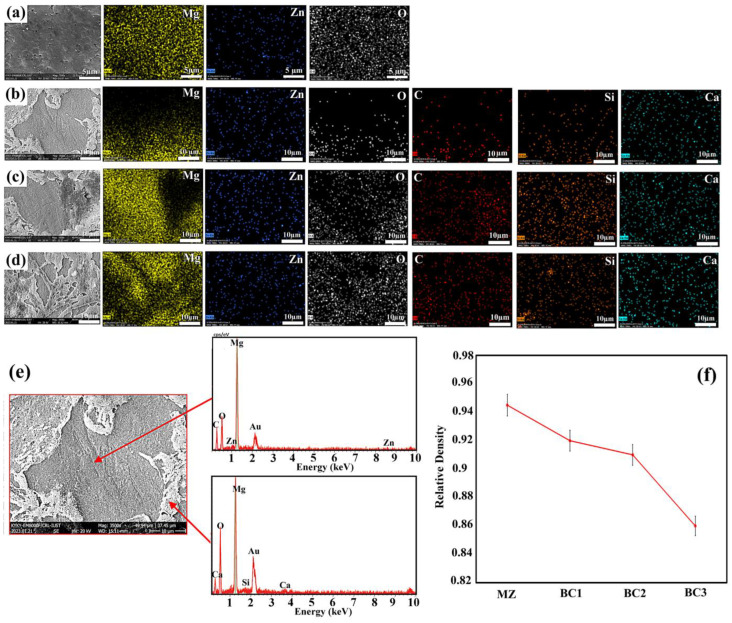
SEM and map images (**a**) MZ ×7000, (**b**) BC1 ×3500, (**c**) BC2 ×3500, (**d**) BC3 ×3500, (**e**) SEM and point EDS analysis of BC1 composite, (**f**) Relative density curve for samples after SPS process.

SEM images and EDS map spectra of composites sintered using the SPS methods are shown in Figure 4. They show that high volume fraction bioceramic particles with minimal porosity can be successfully fabricated. The composite microstructure in this study shows that the base alloy is isotropically surrounded by dispersed bredigite grains. Also, in Figure 4d, the EDS point test related to the BC2 composite is another way of confirming the explanations. Relative density (experimental density compared to theoretical density) decreases as the number of reinforcing particles increases. This can be attributed to the decrease in compressibility of the samples with increasing Br + CNTs, since the reinforcing particles are much harder compared to the base Mg alloy. Another reason that can be stated for this issue is the preventive effect of reinforcing particles on the sintering mechanism. The high melting temperature of the reinforcing particles causes them to have little tendency to bond with the base alloy, which results in the formation of weak networks. Of course, on the other hand, as is clear from Figure 4f, the increase in reinforcing particles has an inverse relationship with the relative density, and a relatively greater drop was observed in the samples with higher numbers of reinforcing particles.

**Figure 5:**
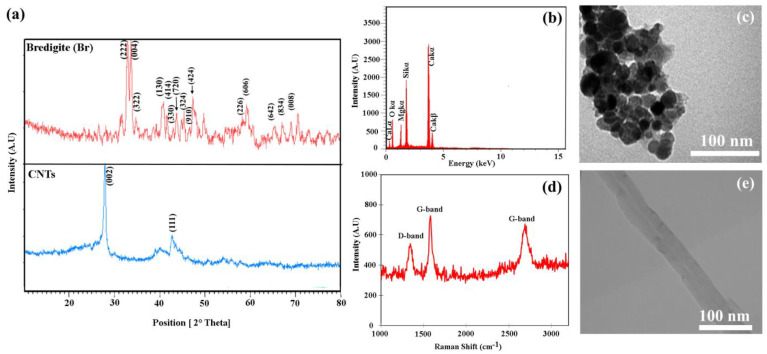
(**a**) XRD pattern for bredigite, CNTs, (**b**) EDS spectrum of bredigite, (**c**) TEM image of bredigite, (**d**) Raman spectrum of pure CNTs, and (**e**) TEM image of CNTs.

According to the X-ray diffraction spectrum (Figure 5a), the crystallinity of the CNTs can be affirmed by observing the peak in the XRD pattern [1]. Additionally, in the X-ray diffraction pattern the synthesized characteristic peaks related to the bredigite powders were marked [2].

The EDS spectrum diagram Figure 5b shows that all essential elements such as Si, Mg, Ca and O are present in the structure. In the TEM image of the bredigite powder in Figure 5c, a spherical morphology can be seen. Each agglomerate is composed of multiple bredigite crystals with a particle size range of 20–30 nm. The Raman spectrum for MWCNTs (Figure 5d) shows the existence of D band (1353 cm^−1^), G band (1575 cm^−1^) and D’ band, the second-order new D band (2696 cm^−1^), which is a distinctive feature. This confirms that CNTs are multi-walled [3].

The diameter of a CNT can usually be determined by directly measuring the distance between the two dashed lines in its TEM image [4]. The TEM image of the CNTs following the dispersion procedure applied in this research is presented in Figure 5e. Compared to the pre-dispersion stage, there were almost no CNTs agglomerates, and the diameter size of individual CNTs ranged from 50 to 60 nm.

**Figure 6:**
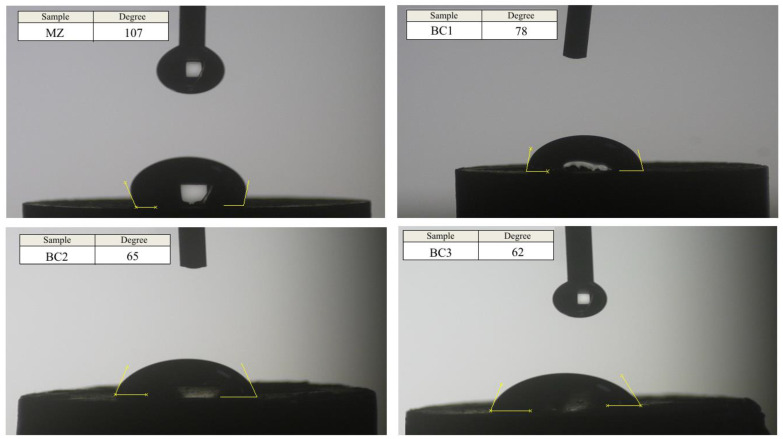
Contact angle for MZ, BC1, BC2 and BC3 composites after SPS process.

According to Figure 6, the contact angle of a drop of liquid with the bulk surface for MZ, BC1, BC2 and BC3 samples is 107, 78, 65 and 62 degrees, respectively. The presence of Br-CNTs reinforcing particles in the background phase reduces the contact angle of water due to their hydrophilic properties, and since silicate bioceramics are hydrolyzed in physiological environments, this results in increased water absorption [5,6]. On the other hand, the presence of carbon elements causes the creation of functional groups including carboxyl (COOH), carbonyl (C=O) and hydroxyl (O-H), which lead to hydrophilicity. As the biocompatibility of Mg-based implants improves, the hydrophilicity also improves [7].

### 3.2. Mechanical Properties

**Figure 7:**
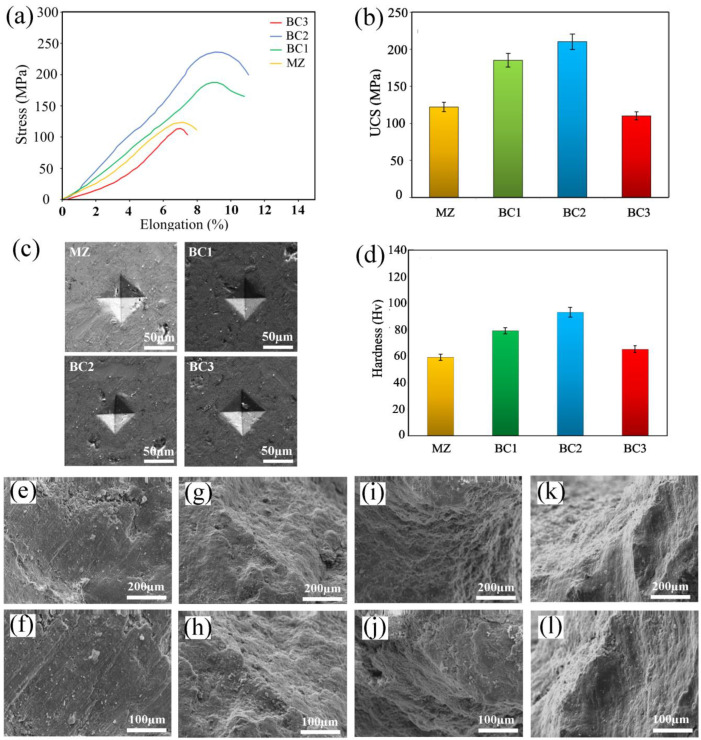
(**a**) Compressive curves (**b**) ultimate compressive strength (UCS) diagram, (c) surface morphology after deformation by press indentation ((**d)** the value of microhardness, (**c**–**j**) SEM images of the fracture surface of the composites after the compressive strength test: (**e**) MZ ×150; (**f**) MZ ×300; (**g**) BC1 ×150; (**h**) BC1 ×300; (**i**) BC2 ×150; (**j**) BC2 ×300; (**k**) BC3 ×150; (**l**) BC3 ×300.

In clinical applications, the mechanical behavior of biomaterials is of great importance, especially when it comes to replacing hard bone tissue [8,9,10]. Compressive strength testing is very important to verify the effectiveness of the mechanical behavior of Mg-based implants [11]. Figure 7a,b shows the compression test results for Mg-based implants with different degrees of reinforcement. Interestingly, the effects of strengthening on the properties of Mg composites are variable. It was observed that the presence of Br-CNTs reinforcements in composites BC1 and BC2 increases the compressive strength, and specifically, BC2 composites have the highest UCS. This may be due to the irregular multi-edge bredigite particles breaking the oxide film on the surface of the Mg powder particles by pressing the matrix [15,16]. This happens during the SPS process, strengthening the bond and making it more resistant to failure under load [15,16]. the presence of CNTs as nanofillers in composites with unique properties, such as very high Young’s modulus, can also accelerate charge transfer from matrix to reinforcement [17,18,19,20,21]. On the other hand, according to the Orowan mechanism, the reinforcing particles act as a barrier to the free dislocations movement in the matrix, increasing their resistance to deformation [11]. With the presence of larger amounts of reinforcement (BC3), we see a decrease in mechanical properties, which can be attributed to agglomeration.

Figure 7c,d shows the micro-hardness values of the samples and the surface morphology of the indentations of the various samples as a result of the hardness test. It was observed that the hardness value amplified with increasing of CNTs-Br reinforcement in comparison to the MZ matrix alloy. The hardness values of MZ, BC1, BC2, and BC3 were HV 59 ± 2.3, HV 79 ± 3.1, HV 93 ± 3.6, and HV 65 ± 2.4, respectively. BC2 composites had the highest hardness compared to other composites and also showed a significant increase compared to matrix alloy.

This improvement in strength—which is attributed to the favorable interaction of the metal matrix alloy through synergy with bioceramic particles and base carbon elements as fillers, and due to the homogeneous distribution of particles that can cause effective force transmission—has also been reported by other researchers [12,13,14,15]. On the other hand, with the increase of reinforcing particles, the intermolecular attractive force between the particles increases and the particles tend to agglomerate, which reduces the strength of the nanocomposite [13,14].

Figure 7e–l shows the morphology of the compressed fracture surface of the MZ/Br-CNTs composite. Almost all nanocomposites exhibited fracture angles of about 45 degrees with respect to the stress axis. In addition, some shear properties consistent with standard brittle fracture patterns were observed in the MZ/Br-CNT nanocomposites.

The deformity of Mg composites was controlled by twinning shear bands. Consequently, shear banding resulted from work-hardening behavior and non-uniform deformation [16].

Composites that break due to shear band deformation have a high work-hardening rate. This form of shear banding has been reported in Mg compression fracture surfaces, and the presence of tensile twinning has been indicated as the dominant plastic deformation when the material is exposed to compressive loading [17,18]. A further barrier to slide diffusion is the dominance of tensile twin diffusion in the Mg matrix by Br and CNTs. This was especially true at the boundaries between Mg particles [15,19,20]. The increase in shear band detected on the fracture surfaces of MZ/Br-CNTs composites can be ascribed to the extra flexible compressive fracture behavior and mixed fracture mode surfaces in the nanocomposite containing moderate amounts of Br and CNTs (Figure 7g-j) [17,19]. On the other hand, the presence of a large content of Br-CNTs reinforcements in the composite matrix can cause accumulation in the composite (Figure 7k,l).

**Figure 8:**
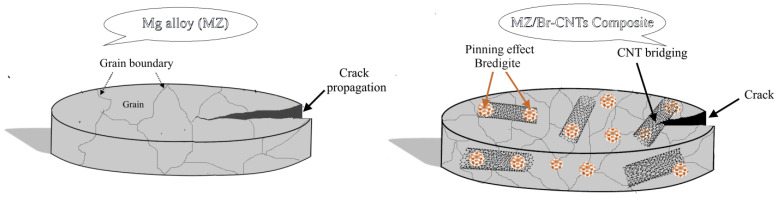
Schematic of the simultaneous strengthening mechanism of Br-CNTs during tension.

The scheme in Figure 8 shows that the presence of bredigite nanoparticles can play an important role in pinning CNTs to grain boundaries due to the dendritic surface and the resulting stronger bonds. On the other hand, smaller grain sizes were created in nanocomposites, which led to grain refinement [21]. Therefore, when stress is applied to the composite, CNTs can act as a bridge and partially prevent crack propagation [21].

### 3.3. Degradation Rate Assessment

**Figure 9:**
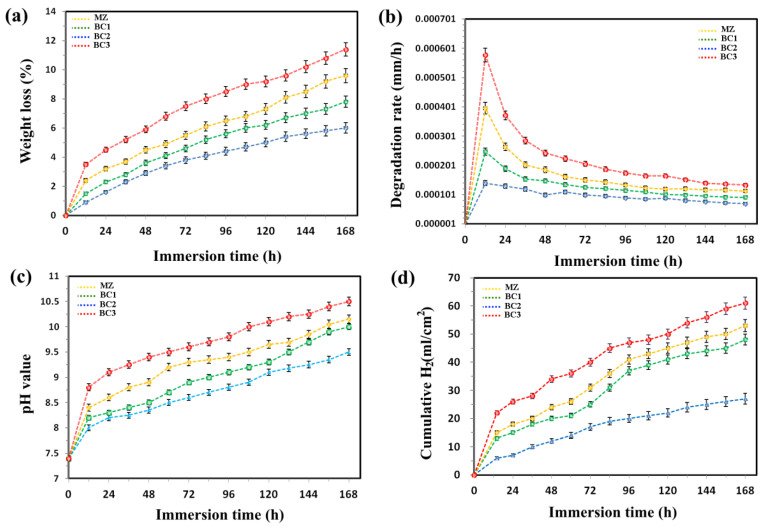
Graphs of (**a**) weight loss, (**b**) degradation rate, (**c**) pH values and (**d**) H_2_ values associated with various composites subjected to immersion test.

As soon as the sample was introduced into the SBF solution, hydrogen bubbles began to be released, indicating the initiation of a reaction between the sample and the solution and the initiation of weight loss by the sample. According to the composite weight loss graph under test Figure 9a, the lowest degradation rate was associated with BC2 composites with a half reduction (Figure 9b). Interestingly, the weight loss was significantly increased in samples with high amounts of reinforcement (Figure 9a). This may be a direct consequence of the accumulation of Br-CNTs in the matrix and deterioration as a result of galvanic corrosion [22,23,24]. The pH of the SBF solution was also monitored throughout the degradation experiment to distinguish the degradation products (Figure 9c). Slow increases in pH were detected for all combinations of substances examined. The increased alkalinity of the SBF solution is probably because of the diffusion of OH- ions and the deposition of Mg(OH)^2^ on the implant surface [25,26]. Here, we showed that the BC2 composite sample significantly reduced H2 emission from 53 ± 2.2 to 27 ± 1.8 mL/cm^2^ compared to the base alloy (Figure 9d).

**Figure 10:**
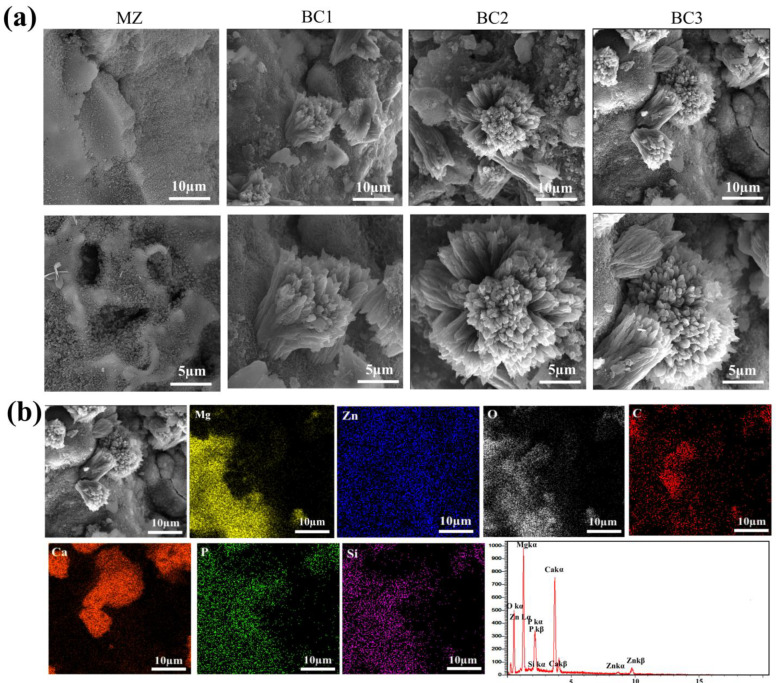
(**a**) SEM images of various composites after 21 days of exposure to SBF solution in magnification ×5000 and ×10,000, and (**b**) SEM and EDS analysis of BC2 composites after exposure to SBF solution.

SEM and EDS analysis (Figure 10) was performed to get a better understanding of surface morphology and degradation results. It is clear that the surface of the MZ sample was completely corroded and exfoliated, with corrosion products covering the surface. Pitting corrosion that originated from the sidewalls and surface of the sample and progressed toward the core caused the collapse of the MZ sample after 14 days (Figure 10a; MZ). Bredigite particles mainly limit the penetration of corrosive solutions by blocking the pore paths of the matrix phase and also by creating a protective surface layer [27]. The SEM image shows that the Br-CNTs reinforced composite has less voids. In addition, the SEM image and corresponding EDS map spectra clearly show the presence of calcium, and especially phosphorus, which originates from the SBF solution, on the surface of the composites. Mg^2+^ and Ca^2+^ ions were generated due to the existence of bredigite particles [28]. Mg^2+^ and Ca^2+^ were adsorbed on the surface of the sample, and the silicon-rich region is shown on the map (Figure 10b). An ability of the composites to facilitate the formation and absorption of salts containing bioactive CaP. B. Hydroxyapatite (HA) was detected.

**Figure 11:**
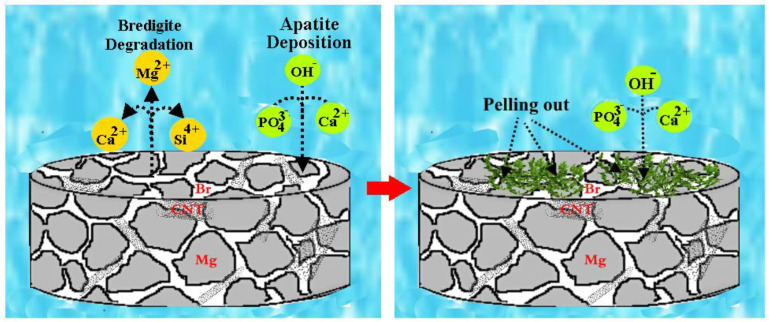
Schematic diagram of bridigite fracture and apatite formation in SBF solution.

Throughout the disbanding of bredigite particles, Ca^2+^ were preferentially released over silicon ions due to exchange with H^+^. Therefore, most of the Si in bredigite remains in the inactive portion, creating a negatively charged surface with the functional group (≡Si–O-) which electrostatically attracts Mg^2+^ and Ca^2+^. Consequently, when Mg is present in the microenvironment near the corroded surface, it will be attracted by the negatively charged (≡Si–O-) functional groups and then permeate through HA lattice ion exchange with Ca^2+^. Then, a Ca-deficient, Si- and Mg-comprising HA is made, which is more bioactive than the stoichiometric HA (Figure 11) [7,27].

In addition, deposition of apatite in the Mg matrix can be facilitated by oxygen-rich groups in carbon materials such as CNTs. This is because the carbonaceous material provides a useful site for the nucleation of hydroxyapatite, which can lead to the formation of a dense apatite layer and thus can prevent further penetration of SBF [29]. Moreover, previous studies have shown that CNTs can act as bridges when used as composite reinforcements due to their thread-like appearance [30], and prevent the oxide layer from peeling off the alloy, which in turn can improve corrosion resistance [30,31,32].

### 3.4. Antibacterial Evaluation

**Figure 12:**
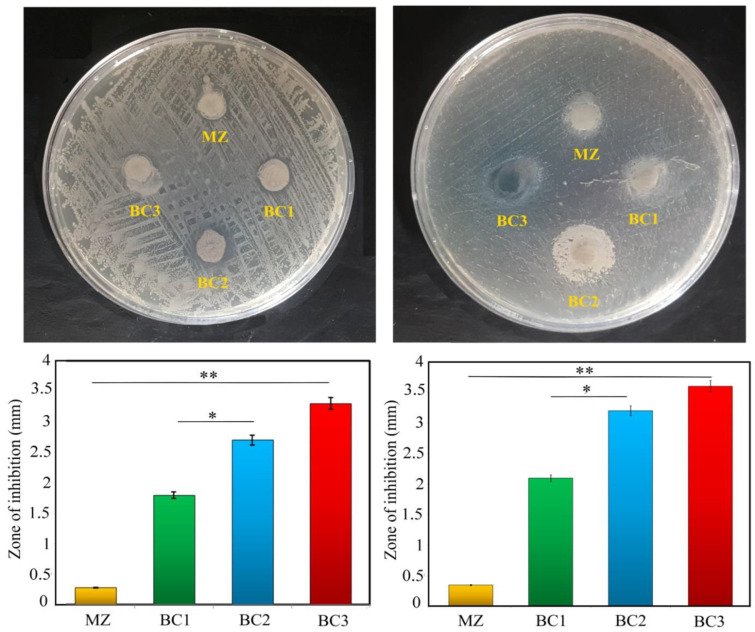
Antibacterial activity for different composites by disc diffusion method against Gram -positive bacteria and Gram-negative bacteria (* *p* ≤ 0.05, ** *p* ≤ 0.01).

Assessment of antibacterial activity of different MZ/Br-CNT samples was done by exposing the composites to Gram-positive and Gram-negative bacteria, namely E. coli and S. aureus, and by measuring the diameter of non-growth of bacteria (Figure 12).

As is evident from Figure 12, bacterial growth continues around the MZ samples, but stops around all nanocomposites containing Br-CNT. In addition, it can be seen that near BC3 on the agar plate there is a wider inhibition zone compared to the halo zone, which has not grown near BC1 after 24 h. In other words, increasing Br-CNT increased the inhibition zone or growth halo of the composite. Thus, the bacterial inhibition zones for Escherichia coli and Staphylococcus aureus ranged from 0.25 to 3.3 mm and 0.36 to 3.6 mm, respectively.

Previous scientific studies have shown that the antibacterial activity of calcium silicate ceramics, including bredigite, is due to the exchange of alkali ions with protons from the aqueous medium, and hydroxyl ions due to their inhibitory and antibacterial properties [33]. It has also been found that the antibacterial property of bredigite has an inverse relationship with the particle size, because smaller particles are more likely to release calcium ions, which leads to an increase in the pH value [34]. In other words, two factors can increase pH in the presence of bredigite ceramics: Ca ion concentration, and particle size. In solution, the concentration of Ca ion in silicate ceramics is much higher than other cations such as Mg ion. In contrast, it has been demonstrated that Mg ion has nothing to do with antibacterial activity [35]. Therefore, compared to Mg ions, Ca ions have a pronounced bactericidal effect and are a major factor in increasing solution pH [36].

According to some studies, CNTs may also show complete antibacterial activity. The surface-to-volume ratio of carbon nanomaterials increases with decreasing size, allowing microorganisms to strongly adhere to membranes and cell walls, leading to more effective working performance. Therefore, their size plays a significant role in the inactivation of microorganisms. This mechanism is based on the capability of CNTs to attach to microorganisms and disturb their cell membranes, morphology, and metabolic processes [37,38,39,40]. The bacteriostatic activities of CNTs have been demonstrated to derive from their capability to damage microbial cell membranes and make bacterial cell death upon direct contact [37,41,42].

### 3.5. Cellular Compatibility

**Figure 13:**
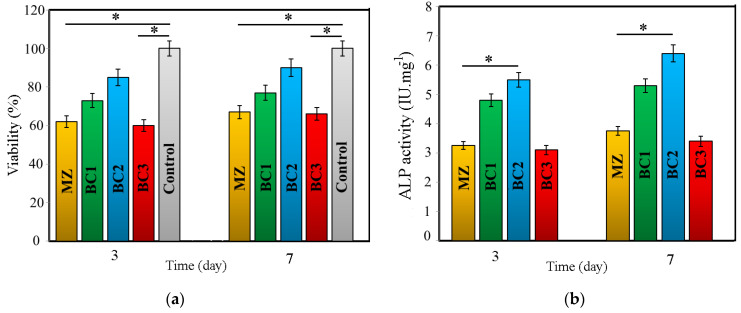
(**a**) Cell viability and (**b**) ALP activity for different composites (* *p* ≤ 0.05).

Figure 13 displays the survival percentage of bone cells after three and seven days of culture. The MG63 cell culture results showed that the percentage of cells increased with increasing cell culture time, indicating that none of the samples were toxic. Furthermore, as shown in Figure 13, there was a considerable difference in the survival rate of MG63 cells between the BC2 sample extract and the remaining samples, indicating that the survival rate was high.

Wu et al. [43] found that the ionic products resulting from lysis of bredigite can increase cell proliferation. Si release played a very important role in ossification. Salicylic acid (derived from the dissolution of bredigite) can increase the production of type 1 collagen, and the release of salicylic acid into the environment increased the ability of osteoblasts to survive and proliferate [43].

Alkaline phosphatase (ALP) activity, commonly used as an early marker of osteoblastic differentiation, was measured on days 3 and 7. The results were similarly shown in Figure 13 for BC2 extract compared to MZ extract.

## 4. Conclusions

In this study, mechanical alloying and semi-powder metallurgy processes were used in conjunction with spark plasma sintering to produce high density MZ/Br-CNTs composites. Adding Br and CNTs nanofiller to the MZ matrix alloy at the same time improved mechanical strength. In addition to having adequate bioactivity, the creation of a single Ca-P film also protected the surface, and a controlled corrosion rate was observed. The combination of Br and CNTs with a MZ matrix alloy showed an effective synergistic effect on antibacterial performance. Therefore, the MZ/Br-CNTs composite is promising for biodegradable implant applications due to its excellent mechanical and antibacterial properties, acceptable cell compatibility, and relatively low corrosion rates in vitro conditions.

## Figures and Tables

**Table 1:** Designation and nominal compositions of (Mg-3Zn)_x_/Br_x_-CNTs_x_ nanocomposites.

Material Designation	Formula	Composition, wt.%			
		Mg	Zn	Br	CNTs
MZ	Mg-3Zn	97	3	0	0
BC1	(Mg-3Zn)_94.5_/Br_5_-CNTs0.5	91.66	2.83	5	0.5
BC2	(Mg-3Zn)_89_/Br_10_-CNTs_1_	86.33	2.67	10	1
BC3	(Mg-3Zn)_83.5_/Br_15_-CNTs1.5	80.99	2.50	15	1.5

## Data Availability

All data provided in the present manuscript are available to whom it may concern.
